# Responsiveness of the healthcare system in the Kingdom of Saudi Arabia: evidence from a nationally representative survey

**DOI:** 10.1186/s12913-022-08779-5

**Published:** 2022-12-14

**Authors:** Zlatko Nikoloski, Taghred Alghaith, Christopher H. Herbst, Mariam Hamza, Quds AlSaffer, Mohammed Alluhidan, Di Dong, Reem Alsukait, Latifah Alqasem, Nahar Alazemi

**Affiliations:** 1 Department of Health Policy, London School of Economics and Political Science, London, UK; 2 Saudi Health Council, Riyadh, Kingdom of Saudi Arabia; 3 World Bank, Washington DC, USA; 4 Department of Community Health, College of Applied Medical Sciences, King Saud University, Riyadh, Saudi Arabia; 5 Saudi Arabian Monetary Authority, Riyadh, Kingdom of Saudi Arabia

**Keywords:** Responsiveness, Saudi Arabia, Inpatient, Outpatient, Determinants

## Abstract

**Background:**

Responsiveness is one of the widely used metrics in assessing the performance of healthcare systems. An analysis of the determinants of health care demand and supply and how the Saudi health system responds to the needs of patients (inpatient and outpatient) is needed; hence the need for this study.

**Methods:**

We analysed data from the Saudi Health Systems Responsiveness survey – a nationally representative survey of 10,000 households interviewed in 2017. Using this dataset, we descriptively analysed the level of responsiveness of inpatient and outpatient services (using the standard World Health Organization (WHO) responsiveness dimensions). Based on a logit modelling approach, the relationship between responsiveness and its key determinants was analysed in terms of healthcare demand and supply.

**Results:**

Over four fifths of respondents are satisfied with the level of inpatient and outpatient responsiveness. Furthermore, we find that those in bad health tend to show lower levels of satisfaction with inpatient and outpatient care. We also find some evidence that age, gender, and to some extent nationality act as correlates of health system responsiveness. Specifically, we find evidence that Saudi nationals are less satisfied with health services compared to foreign nationals.

**Conclusion:**

Based on these findings improving the responsiveness of public healthcare facilities would need to be prioritized. Focusing on patients in worse health and lower socio-economic status should also be one of the main priorities.

**Supplementary Information:**

The online version contains supplementary material available at 10.1186/s12913-022-08779-5.

## Introduction

Historically, a few metrics have been used in assessing the performance of national healthcare systems. Building on the role that healthcare plays in improving health outcomes, analysts have resorted to using mortality indicators when assessing the performance of health systems [1]. Most recently, access to healthcare and equity of access to healthcare have also been used as indicators when measuring the performance of healthcare systems [2]. In addition, over the last few decades, experts have been using out-of-pocket healthcare expenditure as an indicator in measuring how well a healthcare system is faring. In particular, analysts have been focusing on analysing if using healthcare is associated with excessive healthcare payments, thus pushing some households below the poverty line [3].

Over the last 20 years, responsiveness of the healthcare system has emerged as an additional indicator in assessing health systems’ performance [4]. It has been documented that responsiveness has a direct impact on health outcomes and thus, it is closely correlated with some of the performance indicators mentioned above. For example, studies found that satisfied patients tend to adhere to recommended courses of treatment and return for required follow-up visits [5]. Moreover, as the current literature suggests, patients who are satisfied are more likely to adhere to the prescribed medical treatment, thus improving both, compliance as well as continuity of care [5].

Responsiveness is defined as the non-medical aspects of the treatment of individuals such as the treatment environment or the interpersonal relationship between doctor and patient [6]. The concept of responsiveness is particularly important as it could increase both, adherence to treatment as well as the use of preventative healthcare services [7]. The interpersonal, doctor-patient relationship, while also paying attention to the role of gender norm, is at that core of the WHO definition [8].

The concept of responsiveness focuses on 7 aspects ranging from confidentiality between doctor and patient to level of cleanliness of the healthcare facilities [9]. All seven aspects are subjective and cover different non-clinical dimensions of seeking healthcare [10]. With that said, this is the most comprehensive definition, and thus it is wider than other, previously used definitions, mainly focusing on the doctor-patient interpersonal communication [11, 12].

Patient expectations are the crux of the concept of responsiveness [13]. More specifically, the responsiveness is the non-clinical outcome of the process of seeking care, whereby the patient compares the experience against the expectations that he/she had [13]. These expectations, in turn, are influenced by the patient’s characteristics such as: age, gender, socio-economic status or previous experience with the healthcare system [14]. The interplay of the demographic characteristics and previous experience, in turn, shapes the expectations, which, if acted upon, increase the level of the patient’s satisfaction [15].

In Saudi Arabia, the concept of responsiveness and its correlates have been studied [16, 17]. However, most of the studies are small scale, involving either one hospital or healthcare centre [16, 17]. In other words, to date, to the best of our knowledge, no study has analysed the concept of responsiveness using a nationally representative data. In addition, while most of the studies have focused on finding a link between patients’ demographic and socio-economic characteristics and their satisfaction with healthcare [18], there is very little knowledge on the differences in responsiveness in private vs. public healthcare facilities. The existing evidence from these small scale studies finds that gender, income status and level of education are the most important socio-demographic correlates of responsiveness [19].

Against this background, the objective of this research effort are two-fold: (i) to descriptively analyse the level of Saudi’s health system responsiveness; and (ii) to use the standard logit modelling approach in order to distil the main demographic and socio-economic correlates of responsiveness. By meeting these objectives, the paper contributes to the literature in a few crucially important ways. First, it sheds light on some of the demographic and socio-economic correlates of patient satisfaction mentioned above. Second, and building on the previous point, the study is comprehensive in that it takes into account satisfaction with both, outpatient and inpatient healthcare services. Third, and more importantly, it also provides a further understanding of the public/private split of health systems responsiveness in Saudi Arabia.

## Methodology

### Data

For the purpose of this research endeavour, we relied on the Saudi Health Responsiveness survey. It is a nationally representative survey covering all 13 provinces in the country carried out in 2017. The survey consists of a number of modules covering various aspects of the healthcare system: health status of respondents (subjective assessment if respondents are in good or bad health), number of chronic illnesses (including the most common types of chronic illnesses such as diabetes), and utilization of inpatient and outpatient healthcare services (both, if the respondents have sought inpatient or outpatient healthcare service as well as the number of times healthcare was sought) as well as the satisfaction with inpatient and outpatient healthcare. The modules on outpatient and inpatient satisfaction with healthcare include the standard WHO dimensions of responsiveness enumerated in the introduction section above: immediate attention, dignity, communication, confidentiality, surrounding, independence, choice and, in the case of inpatient healthcare utilization, social support. The survey also contains modules on home care and satisfaction with home care. The survey contains data on 10,000 households. The data were collected face to face where one household member was randomly selected to respond to questions from modules as described above. Finally, the survey includes weights which are nationally representative of age, gender and nationality.

### Analytical approach

In doing the analysis we followed the following approach: (i) first each responsiveness dimension was summarised descriptively in order to show the level of patient satisfaction per different dimension (in the following section, we further elaborate on the definition of different responsiveness dimensions); (ii) second, each of the categorical variables representing a responsiveness dimension was transformed into a binary variable taking value of 1 (if the respondent answered 3 or 4 on the Likert scale) and 0 otherwise. The dummy variables were then used as dependent variables in a standard logit model on correlates of responsiveness.

### Dependent variables

In studying the responsiveness of the healthcare system in Saudi Arabia we rely on two modules from the survey: outpatient care and inpatient care. Both modules contain questions on various dimensions of responsiveness, which we use as our main variables of interest corresponding to the WHO framework: immediate attention, dignity, communication, independence, confidentiality of information, choice of provider and quality of surrounding. All of them are measured on a 0–4 Likert scale (details of question wording and variable creation is provided in the Supplementary material).

### Independent variables

The survey contains the standard set of demographic and socio-economic variables which are crucial in conducting a standard logit model presented in the methodology section above. The following independent variables were used: age (a categorical variable with seven ten-year period categories), gender (a dummy variable capturing if the respondent is male or a female), education (a categorical variable capturing the education level starting from not being able to read and write to postgraduate education), nationality (a dummy variable capturing if the respondent is a Saudi national or an expat), self-rated health status (a categorical variable with five categories: very bad, bad, average, good, very good), number of chronic conditions (a categorical variable with the following categories: no chronic illness, one, two, three, four, and five or more chronic illnesses). Details of the survey questions and their transformation into the variables of our models is available in the Supplementary material.

In addition, we also explore the supply side of the health system responsiveness and we do so by including: dummies for the various regions and second, the place where the healthcare was sought (e.g. public, private etc). As there are a few healthcare facilities in the public domain (e.g. those associated with the Ministry of Health, those associated with Ministry of Defence etc) we explore the responsiveness of the public sector only and report the results as an appendix (further details are provided in results section below).

Finally, the survey contains a mini-module on patient treatment experiences which are instrumental in studying the responsiveness of the healthcare system in Saudi Arabia. More specifically, a module on patient mistreatment was included in the survey which asked respondents if they have been mistreated based on a few socio-economic and demographic characteristics: nationality, social background, lack of insurance, skin colour, gender, language, religion, health status and weak physical condition. This question was used to construct a few dummy variables capturing something that we name as ‘subjective socio-economic characteristics’ and we used these in order to provide further robustness checks to our findings when using objective assessment of socio-economic characteristics.

### Logit modelling

Against this background, if we assume a linear model, the main variables of interest (i.e. various dimensions of responsiveness) can be analysed by regressing them (yi) on a vector of k socio-economic and demographic variables (xk).

The equation would be as follows:

$${y}_i^{\ast }=\alpha +\sum_k{\beta}_k{x}_{k,i}+\varepsilon i\textrm{with}\ \textrm{i}=1,\dots \textrm{N}\kern0.5em (A).$$

Where α, β = parameters and ε_i_ = error term.

Assuming that y_i_^*^ in eq. (A) is a latent variable, the logit model is written as:

$$\left\{\begin{array}{c}1\ if\ {y}_i^{\ast }>0\\ {}0, otherwise\end{array}\right.$$

In addition to this and as a robustness check, we have also constructed a simple responsiveness index for outpatient and inpatient care (an additive index of all of the dimensions above) [20]. While there are some caveats to combining various dimensions of responsiveness under one roof (e.g. some of the dimensions follow different wording) constructing the index could allow us to further establish the robustness of our findings. The details of the construction of the index as well as the results when using it are provided in the Supplementary material.

The logit model was conducted using the weights provided in the survey clustered on survey strata. All of the analyses were done in Stata 14.

## Results

Two-fifths of respondents have one or more chronic diseases. In terms of self-assessed health, the majority of the sample appears to have good or very good health (89%). In addition, most respondents in the sample are in their 30s or 40s. The majority of respondents have either a high school diploma (28.6%) or a university degree (26.6%). Finally, about two-thirds of the respondents (64%) are Saudi nationals (Table A1).

The general level of outpatient and inpatient responsiveness is high. Apparently, 70% of respondents indicated that the immediate care they received in the outpatient and inpatient settings was good or very good (69.7%). Moreover, responsiveness is even higher in the other dimensions (Table 1).

**Table 1:** Saudi Arabia: responsiveness of outpatient and inpatient care, in %

Outpatient		Immediate attention	Dignity	communication	Confidentiality	Surrounding	Independence	Choice	
	very bad/bad	5.6	1.6	2.8	1.3	5.3	4.7	14.5	
	average	24.4	9.8	14.0	9.1	19.0	17.0	26.4	
	good/very good	70.0	88.6	83.2	89.6	75.7	78.3	59.1	
Inpatient		Immediate attention	dignity	communication	confidentiality	surrounding	independence	choice	social support
	very bad/bad	5.7	1.8	3.0	1.8	5.0	5.3	17.8	1.3
	average	24.6	11.4	17.5	10.3	20.3	17.6	25.6	7.6
	good/very good	69.7	86.8	79.5	87.9	74.8	77.1	56.6	91.1

It appears that the level of responsiveness is higher in private care than in public care. Moreover, there is a significant discrepancy in responsiveness between public and private health care. Specifically, this discrepancy ranges from 6 percentage points for confidentiality to 21.6 percentage points for direct care. In the inpatient setting, the discrepancy also ranges from 8.8 percentage points for confidentiality to 31.7 percentage points for choice (Table 2).

**Table 2:** Saudi Arabia: responsiveness of the healthcare system, public/private split, in %

Outpatient			Inpatient		
	public - % good and very good	private - % good, very good		public - % good and very good	private - % good, very good
Immediate attention	59.3	80.8	Immediate attention	62.6	82.6
dignity	84.6	92.5	dignity	83.5	92.5
communication	77.3	88.4	communication	73.6	87.9
confidentiality	86.3	93.0	confidentiality	84.5	93.3
surrounding	68.1	82.7	surrounding	67.9	86.5
Independence	72.7	83.4	Independence	71.3	86.5
choice	48.3	69.6	choice	47.1	78.8
			social support	89.8	93.7

In addition to the public private split, there is a significant geographic variation in the level of responsiveness. The appendix Table A2 captures the geographic variation in responsiveness by dimension and by region for outpatient and inpatient care. Overall, the findings are consistent across regions and across dimensions suggesting that the level of responsiveness is among the highest in the capital. More specifically, when considering the first dimension of responsiveness (immediate attention) in Riyadh, 86 and 80% of respondents are satisfied with the outpatient and inpatient level of responsiveness – well above the national average figures. On the other hand, regions such as Jazan or Almadinah show a consistently lower levels of responsiveness.

Table 3 shows the responsiveness of outpatient care, while Table 4 shows the responsiveness of inpatient care. First, we find consistent and robust evidence of an association between health status and health system responsiveness. More specifically, patients with multi-morbidity tend to have a lower likelihood of being satisfied with various dimensions of outpatient care. Specifically, patients with five or more chronic conditions are 0.5 times less likely to be satisfied with confidentiality and 0.3 times more likely to be satisfied with the level of independence than patients without chronic conditions. Similar results emerge for the second health status indicator - self-rated health status. In other words, individuals with better self-assessed health status are much more likely to be satisfied with various dimensions of outpatient care. More specifically, individuals with very good health status are two to five times more likely to rate various dimensions of outpatient care as good/very good than individuals with very poor health status. Second, we find consistent evidence for the public/private health care distinction by finding that patients seeking care in public health care facilities are less likely to be satisfied with various dimensions of outpatient care compared to patients seeking care in private health care facilities. The findings for both public and private were statistically significant in all models, with odds ratios between 0.5 and 0.7.

**Table 3:** Correlates of outpatient responsiveness, objective

	Immediate attention	Dignity	Communication	Independence	Confidentiality	Choice	Surrounding
Relative to no chronic illness							
1 Chronic illness	1.164* (0.0930)	1.320** (0.170)	1.108 (0.172)	0.750** (0.104)	1.077 (0.166)	0.689*** (0.0930)	1.114 (0.150)
2 Chronic illnesses	1.082 (0.159)	0.831 (0.117)	0.713 (0.155)	0.631*** (0.100)	0.639** (0.121)	0.659*** (0.0971)	0.916 (0.104)
3 Chronic illnesses	0.898 (0.215)	1.119 (0.158)	0.966 (0.226)	0.512*** (0.0852)	0.797 (0.216)	0.525*** (0.0654)	1.097 (0.274)
4 Chronic illnesses	0.834 (0.255)	1.099 (0.328)	0.762 (0.274)	0.667** (0.127)	0.666 (0.257)	0.528*** (0.119)	1.091 (0.421)
5 Chronic illnesses	0.845 (0.222)	0.652 (0.180)	0.725 (0.250)	0.450*** (0.119)	0.402*** (0.129)	0.488*** (0.105)	0.711 (0.253)
Relative to very bad self rated health							
Bad srh	2.104** (0.740)	2.256 (1.177)	1.653 (0.844)	1.895 (0.996)	1.656 (1.020)	1.151 (0.530)	1.904 (0.940)
Moderate srh	2.647*** (0.804)	2.262 (1.174)	1.649 (0.805)	1.572 (1.051)	1.276 (0.568)	1.593 (0.918)	2.057* (0.818)
Good srh	4.134*** (1.305)	4.474*** (2.185)	2.361** (1.017)	2.200 (1.474)	2.303* (0.984)	1.417 (0.805)	2.785*** (1.060)
Very good srh	5.180*** (1.565)	3.684*** (1.784)	2.910** (1.301)	2.665 (2.003)	1.743 (0.746)	2.547 (1.637)	2.474** (0.948)
Female	0.930 (0.0630)	1.230 (0.159)	0.997 (0.0904)	1.364** (0.172)	1.298* (0.192)	1.298** (0.146)	0.947 (0.0782)
Relative to less than 20 years old							
21 to 30	1.188 (0.181)	0.950 (0.206)	1.108 (0.298)	0.908 (0.289)	0.726 (0.227)	0.714 (0.200)	1.667** (0.416)
31 to 40	1.257 (0.221)	1.202 (0.321)	1.309 (0.297)	0.853 (0.242)	1.046 (0.369)	0.748 (0.223)	1.643* (0.439)
41 to 50	1.354* (0.230)	1.340 (0.347)	1.513* (0.330)	1.178 (0.373)	1.019 (0.368)	1.182 (0.317)	1.814** (0.463)
51 to 60	1.607*** (0.290)	1.558 (0.554)	1.992*** (0.492)	1.444 (0.491)	1.189 (0.395)	1.310 (0.327)	1.874** (0.476)
61 to 70	1.740** (0.395)	1.149 (0.407)	1.837** (0.500)	1.865* (0.626)	1.050 (0.387)	2.037** (0.602)	1.948** (0.628)
Over 71	2.210*** (0.518)	3.202*** (1.287)	2.593*** (0.641)	2.300** (0.767)	1.591 (0.585)	2.406*** (0.690)	2.285** (0.935)
Relative to can’t read and write							
Can read and write	0.614** (0.123)	0.625 (0.206)	0.632 (0.210)	0.867 (0.347)	0.695 (0.183)	0.842 (0.188)	0.767 (0.201)
Primary completed	0.790 (0.174)	0.884 (0.248)	0.810 (0.253)	0.693 (0.192)	1.160 (0.258)	0.715 (0.162)	0.624* (0.152)
Middle school completed	0.734 (0.158)	0.826 (0.251)	0.612 (0.192)	0.856 (0.318)	0.817 (0.148)	1.041 (0.239)	0.630* (0.166)
High school completed	0.700* (0.137)	0.693 (0.213)	0.585* (0.186)	1.021 (0.326)	0.904 (0.151)	1.078 (0.231)	0.532*** (0.127)
University	0.699 (0.154)	0.702 (0.250)	0.588 (0.218)	0.940 (0.389)	0.805 (0.176)	0.982 (0.243)	0.492*** (0.131)
Post-graduate	0.667 (0.186)	2.320 (1.635)	0.740 (0.353)	1.907 (0.820)	1.897 (1.113)	1.449 (0.334)	0.776 (0.337)
Nationals	0.584*** (0.0710)	0.783 (0.206)	0.822 (0.131)	0.824 (0.191)	0.769 (0.170)	0.708 (0.172)	0.507*** (0.109)
Public health centre	0.544*** (0.0449)	0.586*** (0.0769)	0.578*** (0.0768)	0.643*** (0.0778)	0.704*** (0.0941)	0.531*** (0.0522)	0.633*** (0.0648)
N	6846	6845	6845	6843	6841	6841	6841
pseudo R-sq	0.111	0.088	0.087	0.083	0.078	0.094	0.089

Exponentiated coefficients; Standard errors in parentheses

* *p* < 0.1 ** *p* < 0.05 *** *p* < 0.01

The models also control for regional dummies (not reported here)

**Table 4:** Correlates of inpatient responsiveness, objective

	Immediate attention	Dignity	Communication	Independence	Confidentiality	Choice	Surrounding
Relative to no chronic illness							
1 Chronic illness	1.084 (0.183)	1.043 (0.203)	1.109 (0.182)	1.041 (0.230)	1.206 (0.295)	0.886 (0.136)	1.402** (0.225)
2 Chronic illnesses	1.011 (0.212)	0.808 (0.196)	0.787 (0.188)	0.739* (0.133)	0.751 (0.224)	0.691** (0.122)	0.917 (0.150)
3 Chronic illnesses	0.824 (0.221)	0.746 (0.243)	1.059 (0.290)	0.499*** (0.131)	0.599 (0.197)	0.603*** (0.0949)	1.000 (0.249)
4 Chronic illnesses	1.123 (0.360)	0.971 (0.348)	1.232 (0.346)	0.513** (0.155)	0.648 (0.269)	0.631* (0.172)	1.178 (0.295)
5 Chronic illnesses	0.861 (0.318)	0.539 (0.265)	0.697 (0.248)	0.326*** (0.0904)	0.509* (0.197)	0.716 (0.224)	1.165 (0.355)
Relative to very bad self rated health							
Bad srh	2.053* (0.767)	1.328 (0.587)	1.317 (0.508)	1.070 (0.422)	1.403 (0.618)	2.180 (1.135)	2.493* (1.332)
Moderate srh	1.932** (0.528)	1.553 (0.727)	1.407 (0.452)	1.284 (0.576)	1.772 (0.621)	2.427 (1.720)	2.419* (1.237)
Good srh	2.779*** (0.668)	2.539** (1.191)	2.406** (0.831)	1.997* (0.768)	3.248*** (1.281)	3.113* (1.988)	3.525*** (1.641)
Very good srh	2.902*** (0.824)	1.879 (0.819)	2.382*** (0.667)	2.134* (0.942)	3.111*** (1.201)	3.842* (2.836)	4.455*** (2.330)
Female	1.278** (0.122)	1.042 (0.148)	1.157 (0.185)	1.112 (0.137)	1.346 (0.269)	1.193 (0.138)	0.983 (0.153)
Relative to less than 20 years old							
21 to 30	1.074 (0.361)	1.688 (0.765)	0.974 (0.417)	0.810 (0.340)	1.842 (0.828)	0.928 (0.337)	1.294 (0.483)
31 to 40	1.257 (0.369)	1.215 (0.517)	1.069 (0.343)	1.001 (0.350)	1.468 (0.581)	0.868 (0.249)	1.013 (0.332)
41 to 50	1.500 (0.453)	2.033* (0.788)	1.418 (0.429)	1.497 (0.488)	2.130* (0.918)	1.151 (0.334)	1.244 (0.422)
51 to 60	1.402 (0.430)	1.833 (0.680)	1.188 (0.386)	1.560 (0.626)	1.903 (0.829)	1.176 (0.389)	1.220 (0.404)
61 to 70	1.954** (0.646)	1.822 (0.739)	1.301 (0.411)	1.626 (0.648)	2.270 (1.180)	1.913** (0.624)	1.397 (0.488)
over 71	2.450*** (0.794)	2.805** (1.344)	1.574 (0.611)	2.889*** (1.158)	3.042** (1.464)	1.654 (0.679)	1.665 (0.767)
Relative to can’t read and write							
Can read and write	0.893 (0.206)	0.805 (0.243)	0.698 (0.194)	0.620 (0.233)	1.131 (0.381)	0.647 (0.174)	1.175 (0.287)
Primary completed	0.901 (0.152)	0.751 (0.244)	0.836 (0.228)	0.607 (0.232)	1.387 (0.624)	0.740 (0.154)	0.852 (0.208)
Middle school completed	1.061 (0.276)	0.827 (0.313)	0.875 (0.283)	0.577 (0.210)	1.145 (0.487)	0.861 (0.242)	0.866 (0.316)
High school completed	1.031 (0.201)	0.889 (0.303)	0.754 (0.241)	0.705 (0.250)	1.168 (0.423)	0.846 (0.207)	0.783 (0.226)
University	0.739 (0.181)	0.645 (0.266)	0.559 (0.215)	0.469* (0.198)	0.700 (0.307)	0.683 (0.190)	0.473** (0.150)
Post-graduate	1.063 (0.473)	1.737 (1.294)	0.745 (0.432)	0.652 (0.417)	0.929 (0.721)	0.723 (0.329)	0.507 (0.373)
Nationals	0.711 (0.150)	0.746 (0.209)	0.788 (0.172)	1.182 (0.232)	0.733 (0.171)	0.666 (0.201)	0.654*** (0.106)
Public hospital	0.409*** (0.0776)	0.445*** (0.0972)	0.343*** (0.0708)	0.365*** (0.0643)	0.414*** (0.107)	0.414*** (0.0777)	0.356*** (0.0753)
N	2251	2250	2250	2250	2031	2249	2249
pseudo R-sq	0.079	0.081	0.083	0.106	0.095	0.097	0.097

Exponentiated coefficients; Standard errors in parentheses

* *p* < 0.1 ** *p* < 0.05 *** *p* < 0.01

The models also control for regional dummies (not reported here)

In examining the public sectors, we found that responsiveness is higher in health facilities affiliated with other ministries/sectors than in those affiliated with the Ministry of Health (MOH) (Table A3). In particular, we find robust evidence that responsiveness is lower in MOH outpatient clinics. Given the number of patients served by the MOH, capacity reduction could be an effective strategy to improve responsiveness. However, current reforms to increase competition among MOH clusters through an accountable care organization structure are expected to change this.

There is evidence of a relationship between health status and responsiveness. In particular, multi-morbid patients tend to be less satisfied with inpatient care than patients without chronic conditions. Similarly, respondents with better health status tend to be more satisfied with inpatient health care. In addition, respondents with very good health were two to four times more likely than respondents with very poor health to answer “good/very good” to several dimensions of inpatient care. Again, we find consistent evidence of the split between the public and private sectors concerning inpatient care (Table 4). Individuals who received inpatient care in a public facility were 0.3 to 0.4 less likely to be satisfied with various dimensions of responsiveness than individuals who received care in a private facility (Table 4). Apparently, responsiveness was lowest in hospitals affiliated with the Ministry of Health (Table A4). This is also likely due to the capacity and percentage of the population covered by the Ministry of Health and is likely to change with ongoing reforms.

### Subjective assessment of socio-economic characteristics

In addition to objective measures of socioeconomic status, we also used a module on patient mistreatment as a robustness check. Given some of the income variable’s problems, the module on patient mistreatment may be useful in illuminating the nexus between socioeconomic status and responsiveness (Tables 5 and 6).

**Table 5:** Correlates of outpatient responsiveness, subjective

	Immediate attention	Dignity	Communication	Independence	Confidentiality	Choice	Surrounding
Have you been treated badly because of							
Nationality	0.936 (0.306)	0.905 (0.360)	1.188 (0.474)	1.641* (0.487)	1.262 (0.506)	1.007 (0.275)	0.767 (0.220)
Social background	0.519*** (0.106)	0.330*** (0.0878)	0.304*** (0.116)	0.394** (0.143)	0.414*** (0.134)	0.414*** (0.138)	0.680 (0.191)
Lack of insurance	0.881 (0.165)	0.651* (0.152)	0.813 (0.185)	0.865 (0.174)	0.731 (0.199)	0.796 (0.177)	0.698* (0.138)
Skin colour	0.636* (0.167)	0.233*** (0.0604)	0.592 (0.197)	0.568 (0.307)	0.321*** (0.101)	0.698 (0.293)	0.791 (0.279)
Gender	1.025 (0.386)	1.124 (0.216)	0.716 (0.228)	1.261 (0.452)	1.003 (0.347)	1.042 (0.386)	0.763 (0.290)
Language	1.020 (0.158)	1.006 (0.312)	0.713 (0.160)	0.875 (0.190)	0.930 (0.360)	0.767 (0.192)	0.535*** (0.0933)
Religion	1.765 (0.966)	2.045 (1.035)	1.961 (1.130)	0.979 (0.305)	0.992 (0.486)	0.974 (0.377)	1.207 (0.647)
Health status	0.702 (0.163)	0.593 (0.189)	0.802 (0.257)	0.813 (0.209)	0.692 (0.163)	0.624* (0.158)	0.930 (0.255)
Weak physical conditions	0.790 (0.146)	0.813 (0.183)	0.777 (0.145)	0.629* (0.159)	0.858 (0.194)	0.590*** (0.114)	0.841 (0.175)
N	6913	6912	6912	6911	6909	6909	6909
pseudo R-sq	0.103	0.087	0.088	0.071	0.070	0.077	0.084

Exponentiated coefficients; Standard errors in parentheses

* *p* < 0.1 ** *p* < 0.05 *** *p* < 0.01

Models also control for nationality, place of seeking care and regional dummies (not reported here)

**Table 6:** Correlates of inpatient responsiveness, subjective

	Immediate attention	Dignity	Communication	Independence	Confidentiality	Choice	Surrounding	Social support
Have you been treated badly because of								
Nationality	0.616* (0.168)	0.789 (0.328)	0.720 (0.263)	0.510** (0.145)	0.629 (0.274)	1.171 (0.471)	1.257 (0.411)	0.604* (0.157)
Social background	0.452*** (0.126)	0.580 (0.209)	0.425** (0.179)	0.534 (0.256)	0.387** (0.168)	0.507* (0.192)	0.260*** (0.0758)	0.371*** (0.116)
Lack of insurance	0.717 (0.178)	0.375*** (0.106)	0.517** (0.136)	0.416*** (0.127)	0.544* (0.180)	0.657* (0.159)	0.503*** (0.105)	0.656 (0.238)
Skin colour	0.418 (0.246)	0.272*** (0.131)	0.941 (0.467)	1.099 (0.547)	0.221** (0.146)	0.818 (0.431)	0.590 (0.278)	0.258* (0.183)
Gender	1.508 (0.442)	1.331 (0.389)	0.860 (0.438)	1.649 (0.615)	0.954 (0.419)	0.647 (0.222)	0.637 (0.316)	4.775*** (2.186)
Language	1.179 (0.428)	1.648 (0.672)	2.152** (0.824)	0.810 (0.210)	2.099 (1.111)	0.921 (0.301)	0.981 (0.388)	0.726 (0.353)
Religion	0.775 (0.430)	0.727 (0.471)	0.660 (0.309)	1.452 (1.028)	0.911 (0.774)	0.217* (0.199)	0.917 (0.594)	1.269 (0.913)
Health status	0.962 (0.439)	2.194 (1.377)	1.064 (0.528)	0.993 (0.425)	1.011 (0.409)	0.845 (0.309)	1.350 (0.558)	0.835 (0.399)
Weak physical conditions	0.743 (0.246)	0.876 (0.371)	0.755 (0.277)	0.755 (0.204)	0.642 (0.239)	0.744 (0.254)	0.808 (0.214)	0.862 (0.326)
N	2276	2275	2275	2275	2054	2274	2274	2274
pseudo R-sq	0.079	0.079	0.083	0.094	0.088	0.090	0.098	0.049

Exponentiated coefficients; Standard errors in parentheses

* *p* < 0.1 ** *p* < 0.05 *** *p* < 0.01

Models also control for nationality, place of seeking care and regional dummies (not reported here)

There is a clear relationship between socioeconomic status and willingness to respond to outpatient services (Table 5). Specifically, respondents who were treated poorly because of their social background are 0.3 to 0.5 times less likely to be satisfied with various dimensions of responsiveness to outpatient health care. We also find some evidence that those who were treated poorly because of their skin colour report lower levels of satisfaction. Finally, those who were treated poorly because of health status or poor physical condition tend to be less satisfied with various dimensions of outpatient health care.

The findings on inpatient care responsiveness are reported in Table 6. We find robust evidence that those who have been treated badly based on their social status are less satisfied with various dimensions of inpatient healthcare. Similarly, we also find evidence that those that have been treated badly based on skin colour tend to be less satisfied with, in particular, dignity and confidentiality. While in this case we do not find evidence for a health status/responsiveness link, interestingly, there is some evidence that those that have been treated badly because of lack of health insurance tend to be less satisfied with different dimensions of inpatient healthcare.

### Responsiveness index

The results using the responsiveness index are provided in Appendix Table A5 and A6. First, there is a strong and robust link between health status and inpatient and outpatient responsiveness. More specifically, respondents with 5 or more chronic illnesses are 0.5 times and 0.3 times, respectively, less likely to be satisfied with outpatient and inpatient healthcare received. Furthermore, those with very good health are 5 and 3 times more likely, respectively, to be satisfied with the outpatient and inpatient healthcare. Second, we find an overwhelming evidence for the public/private split with those seeking care in public healthcare facilities less satisfied relative to those seeking care in the private healthcare sector. Finally, and as it was the case for the main analysis on different responsiveness dimensions above, we find evidence for the significance of the other demographic and socio-economic variables (e.g. gender, age, nationality and education).

## Discussion

To our knowledge, this is the first study to examine health system responsiveness using nationally representative data from the thirteen provinces of the Kingdom of Saudi Arabia. Overall, responsiveness is high across different dimensions. It was found that there is some heterogeneity in responsiveness across the country’s thirteen regions. In particular, health status, age, and nationality are the main explanatory variables for responsiveness. In addition, a significant gap was found between the public and private sectors in responsiveness, with patients being more satisfied with the health services they receive in the private health system.

This high levels of responsiveness witnessed by the results is echoed by existing evidence from some of the other countries in the region [21, 22]. Interestingly and consistent with the overall findings from the rest of the region, we find that nationals have lower satisfaction with healthcare services relative to non-nationals. The explanation that has been put forth is that non-citizens are economic migrants, coming from countries where healthcare availability and quality are much lower compared to that of GCC countries, thus pushing up their overall level of satisfaction [23]. Finally, we also find a strong public/private split in responsiveness with responsiveness being higher in the private sector. While this research question is not particularly explored in the Saudi context, there are couple of studies that shed some light on the topic. For example, Al Hawary [24] find that private hospitals score much higher on the responsiveness dimensions relative to public hospitals. Similarly, Alghamdi [25] with his responsiveness research on government hospitals suggests that improving interpersonal skills among doctors should be a priority of government hospitals.

We also find that health status and age are the most important correlates of responsiveness. This result is consistent with existing evidence suggesting that individuals in better health tend to be more satisfied with various interpersonal aspects than individuals in poorer health. This could be due to multiple contacts with the healthcare system and higher expectations of interaction with the healthcare provider among heavier users, i.e., individuals in poorer health [18, 19, 26]. In addition, the higher satisfaction of elderly patients could be due to the fact that most of them used to live in much poorer conditions and therefore have lower expectations that are easier to meet; also, most elderly people have witnessed the rapid economic changes in the country in recent decades and are grateful for the services they receive.

The authorities should prioritize improving the responsiveness of public health institutions, especially those linked to the Ministry of Health, as part of ongoing cluster reforms. In addition, by focusing on lower socioeconomic status (where the prevalence of noncommunicable diseases is higher), the authorities could improve patient adherence to treatment, which would ultimately lead to better health outcomes. In addition, it is important for further research to uncover a potential causal relationship between responsiveness and its correlates. The Health Sector Transformation Program, established for the Kingdom’s vision 2030, with its aim to restructure the health sector in Saudi Arabia towards being a comprehensive, effective and integrated health system, is expected to address some of these observed challenges.

### Limitations

Because this study is a cross-sectional survey, the relationship between responsiveness and its determinants that we find here is correlative in nature. In other words, the relationship does not assert that there is a causal relationship between the various dimensions of responsiveness and their determinants. Second, while we try to be as complete as possible in our use of correlates, some are missing (e.g., income or consumption as a proxy for socioeconomic status). Finally, given the wording of the questions that capture different dimensions of responsiveness, some caution is warranted in interpreting the responsiveness index.

## Conclusions

There are a few findings that emerge from our research efforts. First, we find that those in worse health (captured either by the number of multi-morbidity conditions or self-rated healthcare status) tend to show lower levels of satisfaction with inpatient and outpatient care. Second, based on the subjective measures mentioned above, we find strong evidence that those in lower socio-economic classes are less likely to be satisfied with various dimensions of inpatient and outpatient care. We also find some scant evidence that age, gender and to some extent, nationality, act as correlates of responsiveness of the healthcare system in Saudi Arabia. More specifically, we find some evidence that Saudi nationals show lower levels of satisfaction with healthcare services, relative to non-nationals. Finally, we also find robust evidence for a public/private split in responsiveness in that responsiveness is much higher in private healthcare facilities relative to public ones. More specifically, within the public healthcare services, those associated with the Ministry of Health are associated with the lowest level of responsiveness.

## Supplementary Information

**Additional file 1.**

## Data Availability

The dataset and the accompanying do files are available upon request from the Saudi Health Council.
