# Supplementary material for: Responsiveness of the healthcare system in the Kingdom of Saudi Arabia: evidence from a nationally representative survey
Source: BMC Health Serv Res. 2022 Dec 14;22:1524. doi: 10.1186/s12913-022-08779-5 (PMC9749241; doi:10.1186/s12913-022-08779-5)
Supplement: Supplementary file 1 — Additional file 1. [file 12913_2022_8779_MOESM1_ESM.docx]

*Responsiveness dimension – data cleaning*

Immediate attention. In constructing this dependent variable we rely on the question – “Overall how do you assess the immediate interest in meeting your need for outpatient health services. Immediate attention means having the right time and distance to go from home to a health service delivery place; get urgent care in case of emergency”. Respondents could assess this aspect of responsiveness on the standard Likert scale: (i) 0-very bad; (ii) 1-bad; (iii) 2-average; (iv) 3-good; and (v) 4-very good.

Dignity. For this dimension we relied on the question: “Overall, how do you evaluate the respect for you and the dignity for you while you receive outpatient care. Dignity means: treatment with respect, demonstrate privacy upon clinical examination or disclosure”. As in the case above, here as well, the respondent could answer the question on the standard Likert scale from 0 (very bad) to 4 (very good).

Communication. In constructing this dimension of responsiveness we relied on the question – “Overall, how do you evaluate how your doctor or healthcare provider communicates with you. Communication means: your service provider listens carefully and attentively, your service provider shows you things to understand, the service provider gives you enough time to inquire and questions”. The standard Likert scale was used in answering this dimension of responsiveness ranging from 0 (very bad) to 4 (very good).

Independence. This is another dimension of healthcare responsiveness for which we relied in the following question: “Overall, how would you rate your participation as much as you want to make decisions about your care or treatment. Independence means: your participation in decisions about your treatment and care, obtain your consent before starting treatment or testing by the service provider”. While this question follows the standard Likert scale (0-4), the wording of the categories is slightly different: (i) 0 – it is not permanently possible; (ii) 1- it is not possible; (iii) 2 – sometimes it can be done; (iv) it can be done; and (v) it can be done easily.

Confidentiality of information. In constructing this variable, we relied on the question: “Overall, how would you rate the outpatient method of keeping your information confidential. Confidentiality of information means: keeping your medical history secret, have a conversation with doctors, nurses or other caregivers in a way that other people around you who don’t want to hear you”. As in some of the other dimensions above, here as well the standard Likert scale was used with values ranging from 0 (very bad) to 4 (very good).

Selection. The choice of medical provider was assessed by the following question – “Overall, how would you rate your ability to choose a service provider or health facility. Choice means: ability to choose a doctor, nurse or healthcare provider, ability to go to another health facility. As in the case of independence the Likert scale follows the same structure of the wording of categories: (i) 0 – it is not permanently possible; (ii) 1 it is not possible; (iii) 2 - sometimes it can be done; (iv) 3- it can be done; and (v) 4 – it can be done easily.

Quality of surrounding. Finally, in assessing the quality of surrounding we relied on the following question: “Overall, how do you evaluate the quality of the place and the surrounding environment, for example: space available, seats, clean air and the cleanliness in the healthcare facilities you visited. The quality of the surrounding means: adequate space, seating and ventilation in the waiting room including clean areas, clean bathroom and having healthy and edible food. As in the case of most of the dimensions of responsiveness the standard Likert scale was used in order to answer to this question ranging from 0 (very bad) to 4 (very good).

The same set of dimensions were then repeated on the inpatient module. In addition, the inpatient module contains one extra dimension – social support. In constructing this dimension, we relied on the following question – “In general, how would you rate the extent to which the hospital allows you to communicate with friends and family and continue to engage in social activities and/or religious rites during your hospitalization. Social support means: personal needs of food and other gifts from relative, freedom of religious practice”. As in the two of the dimensions mentioned above, the Likert scale used in answering this question goes from 0 (it can never be done) to 4 (it can be done easily).

*Independent variables – data cleaning and variable creation*

Age. The age of the respondent in the survey is ascertained with the standard question: “How old are you in years”. Based on it, and for an easy interpretation of the findings from the logit model, we have constructed a categorical variable with the following categories: (i) below the age of 20; (ii) from 21 to 30 years of age; (iii) from 31 to 40 years of age; (iv) from 41 to 50 years of age; (v) from 51 to 60 years of age; (vi) from 61 to 70 years of age; (vii) over 71 years of age.

Gender. Based on the question on the respondent’s gender, we have constructed a dummy variable taking a value of 1 if the respondent is a female and 0 if the respondent is a male.

Education. Based on the question: “What is your last level of education”, we have constructed a categorical variable with the following categories: (i) I can’t read and write; (ii) I can read and write; (iii) primary school completed; (iv) middle school completed; (v) high school or equivalent completed; (vi) high institute/university/understudy; and (vii) postgraduate.

Nationality. Based on the question of nationality, we have created a dummy variable capturing the nationals/non-nationals dichotomy.

Self-rated health status. This is an important proxy of the overall health of an individual and it is captured by the question: “Overall, how do you evaluate your health today?”. Based on it we created a categorical variable with the following categories: (i) 0 – very bad; (ii) 1- bad; (iii) 2 – moderate; (iv) 3 – good; and (v) 4 – very good.

Multi-morbidity. The extent to which an individual has numerous non-communicable diseases could also be a reflection of his/her overall health status. Hence, we construct a variable capturing the number of chronic illnesses that a person has. In doing so, we rely on a few questions from the Health Status module asking the respondent to state if being diagnosed with any of the following diseases: (i) high blood pressure; (ii) diabetes; (iii) joint pains or arthritis; (iv) heart disease including atherosclerosis; (v) lung disease including chronic bronchitis; (vi) chest allergies including asthma; (vii) back pain including herniated disc; (viii) frequent migraine headaches; (ix) stroke or cerebral haemorrhage; (x) mental illness including depression and anxiety; (xi) sleep problems; (xii) hearing problems including total or partial hearing loss; (xiii) vision problems including loss of vision, severe vision impairment, colour blindness; (xiv) gastric problems including gastritis, gastric ulcers; (xv) leukemia or cancerous tumours. Based on these questions, we have constructed a categorical variable with the following categories: (a) no chronic illness; (b) two chronic illnesses; (c) three chronic illnesses; (d) four chronic illnesses and (e ) five or more chronic illnesses.

In addition, we also explore the supply side of the health system responsiveness and we do so by including: dummies for the various regions and second, the place where the healthcare was sought (e.g. public, private etc). More specifically in capturing the place of seeking care we rely on two questions, one from the outpatient module and one from the inpatient module. From the outpatient module we rely on the following question: “To which sector the clinic that you visited belong to: 0 – ministry of health; 1- ministry of interior; 2- ministry of national guard; 3- ministry of defence; 4- ministry of education; 5 – King Faisal Specialist Hospital; and 6 – private sector”. Based on this question, we construct a dummy variable for the public/private split which takes value of 1 if the care was sought in any of the public sector healthcare facilities (categories 0 to 5) and 0 if the care was sought in a private healthcare facility. In addition, and as a further robustness check we provide a public sector specific analysis by only focusing on healthcare visits that have taken place in the public sector (categories 0 to 5 above). We report the findings from the analysis in the appendix of this report. Finally, the same question was asked in the inpatient module (i.e. to which sector does the hospital where the hospitalization occurred belong) and we proceeded in using this question in the same way as in the case of outpatient care.

*Index creation*

As mentioned above, in order to simplify the interpretation of responsiveness (rather than looking at various dimensions) we construct a simple additive index. In doing so, we add all of the responses on various dimensions from the Likert scale and divide them by the number of dimensions. The final index is derived by rounding to the nearest integer number (e.g. 3.2 is 3, while 3.8 is 4 etc). Similarly to the rest of the exercises conducted here, the final value of the index was converted into a dummy variable (0-1), taking values of 1 if the index values are 3 or 4 and 0 otherwise. The findings of the exercise are presented in the Appendix Table A4.

Appendix

Table A1. Socio-economic and demographic characteristics of the sample

|  | % | N |
| --- | --- | --- |
| Chronic illnesses |  |  |
| none | 58.0 | 5657 |
| one | 20.5 | 2004 |
| two | 10.6 | 1039 |
| three | 5.6 | 542 |
| four | 2.6 | 258 |
| five or more | 2.6 | 257 |
|  |  |  |
| Self rated health status |  |  |
| Very bad | 0.6 | 55 |
| Bad | 2.1 | 202 |
| Average | 8.4 | 818 |
| Good | 21.4 | 2085 |
| Very good | 67.6 | 6598 |
|  |  |  |
| Female | 26.9 | 2607 |
|  |  |  |
| Age categories |  |  |
| less than 20 | 3.1 | 306 |
| 20 to 30 | 15.2 | 1488 |
| 30 to 40 | 29.6 | 2888 |
| 40 to 50 | 24.7 | 2409 |
| 50 to 60 | 15.5 | 1511 |
| 60 to 70 | 7.4 | 720 |
| over 70 | 4.5 | 444 |
|  |  |  |
| Education attainment |  |  |
| can't read/write | 8.0 | 777 |
| Can read and write | 8.7 | 850 |
| Primary completed | 14.0 | 1360 |
| Middle school completed | 12.5 | 1216 |
| High school completed | 28.6 | 2783 |
| University | 26.6 | 2594 |
| Postgraduate education | 1.7 | 166 |
|  |  |  |
| Saudi Nationals | 63.8 | 6253 |

Table A2. Saudi Arabia responsiveness of outpatient and inpatient services by region, in %

| outpatient |  | Riyadh | Makkah | Almadinah | Alqaseem | Eastern Region | Aseer | Tabouk | Hail | Northern Borders | Jazan | Najran | Albaha | Aljouf |
| --- | --- | --- | --- | --- | --- | --- | --- | --- | --- | --- | --- | --- | --- | --- |
|  | Immediate attention | 86.48 | 62.37 | 40.5 | 77.29 | 72.72 | 69.02 | 47.44 | 73.99 | 41.14 | 46.45 | 77.39 | 77.08 | 66.64 |
|  |  | Riyadh | Makkah | Almadinah | Alqaseem | Eastern Region | Aseer | Tabouk | Hail | Northern Borders | Jazan | Najran | Albaha | Aljouf |
|  | dignity | 95.28 | 84.67 | 75.69 | 86.93 | 92.65 | 90.27 | 71.53 | 86.44 | 67.56 | 82.13 | 94.52 | 89.81 | 88.1 |
|  |  | Riyadh | Makkah | Almadinah | Alqaseem | Eastern Region | Aseer | Tabouk | Hail | Northern Borders | Jazan | Najran | Albaha | Aljouf |
|  | communication | 92.88 | 78.01 | 70.19 | 82.77 | 89.3 | 81.91 | 61.04 | 79.47 | 51.21 | 72.5 | 89.29 | 84.56 | 76.45 |
|  |  | Riyadh | Makkah | Almadinah | Alqaseem | Eastern Region | Aseer | Tabouk | Hail | Northern Borders | Jazan | Najran | Albaha | Aljouf |
|  | Independence | 82.63 | 75.77 | 70.55 | 87.97 | 90 | 78.59 | 76.79 | 73.54 | 39.26 | 59.05 | 76.7 | 69.69 | 46.23 |
|  |  | Riyadh | Makkah | Almadinah | Alqaseem | Eastern Region | Aseer | Tabouk | Hail | Northern Borders | Jazan | Najran | Albaha | Aljouf |
|  | confidentiality | 95.52 | 88.07 | 73.61 | 91.34 | 92 | 89.84 | 74.76 | 85.29 | 66.43 | 82.21 | 93.1 | 95.05 | 86.77 |
|  |  | Riyadh | Makkah | Almadinah | Alqaseem | Eastern Region | Aseer | Tabouk | Hail | Northern Borders | Jazan | Najran | Albaha | Aljouf |
|  | choice | 64.59 | 57.76 | 50.26 | 71.69 | 74.1 | 46.01 | 56 | 51.05 | 19.81 | 38.86 | 44.58 | 52.08 | 34.27 |
|  |  | Riyadh | Makkah | Almadinah | Alqaseem | Eastern Region | Aseer | Tabouk | Hail | Northern Borders | Jazan | Najran | Albaha | Aljouf |
|  | surrounding | 88.08 | 68 | 55.61 | 80.36 | 80.77 | 78.59 | 64.84 | 72.51 | 41.38 | 61.52 | 66.28 | 84.55 | 72.54 |
|  |  |  |  |  |  |  |  |  |  |  |  |  |  |  |
|  |  |  |  |  |  |  |  |  |  |  |  |  |  |  |
| inpateint |  | Riyadh | Makkah | Almadinah | Alqaseem | Eastern Region | Aseer | Tabouk | Hail | Northern Borders | Jazan | Najran | Albaha | Aljouf |
|  | Immediate attention | 80.53 | 67.24 | 59.43 | 78.67 | 77.54 | 67.01 | 48.3 | 73.09 | 51.79 | 51.9 | 57.47 | 78.73 | 53.89 |
|  |  | Riyadh | Makkah | Almadinah | Alqaseem | Eastern Region | Aseer | Tabouk | Hail | Northern Borders | Jazan | Najran | Albaha | Aljouf |
|  | dignity | 89.84 | 85.62 | 75.18 | 85.37 | 95.21 | 87.36 | 80.29 | 80.14 | 66.57 | 81.3 | 92.19 | 92.24 | 73.05 |
|  |  | Riyadh | Makkah | Almadinah | Alqaseem | Eastern Region | Aseer | Tabouk | Hail | Northern Borders | Jazan | Najran | Albaha | Aljouf |
|  | communication | 87.33 | 76.49 | 73.3 | 84.78 | 85.39 | 79.41 | 66.56 | 77.93 | 64.05 | 68.91 | 90 | 81.9 | 55.31 |
|  |  | Riyadh | Makkah | Almadinah | Alqaseem | Eastern Region | Aseer | Tabouk | Hail | Northern Borders | Jazan | Najran | Albaha | Aljouf |
|  | Independence | 87.53 | 73.42 | 72.72 | 85.91 | 83.96 | 71.22 | 78.06 | 75.25 | 40.74 | 68.46 | 79.56 | 69.72 | 37.75 |
|  |  | Riyadh | Makkah | Almadinah | Alqaseem | Eastern Region | Aseer | Tabouk | Hail | Northern Borders | Jazan | Najran | Albaha | Aljouf |
|  | confidentiality | 92.24 | 85.39 | 82.43 | 91.4 | 93.61 | 84.94 | 87.7 | 78.52 | 77.34 | 83.23 | 93.53 | 93.9 | 71.32 |
|  |  | Riyadh | Makkah | Almadinah | Alqaseem | Eastern Region | Aseer | Tabouk | Hail | Northern Borders | Jazan | Najran | Albaha | Aljouf |
|  | choice | 67.12 | 60.62 | 53.46 | 69.78 | 64.25 | 43.37 | 41.45 | 46.1 | 22.81 | 42.67 | 32.42 | 48.69 | 19.33 |
|  |  | Riyadh | Makkah | Almadinah | Alqaseem | Eastern Region | Aseer | Tabouk | Hail | Northern Borders | Jazan | Najran | Albaha | Aljouf |
|  | surrounding | 85.36 | 71.81 | 63.41 | 76.14 | 84.23 | 65.23 | 71.31 | 73.13 | 48.75 | 66.37 | 75.64 | 84.44 | 49.83 |
|  |  | Riyadh | Makkah | Almadinah | Alqaseem | Eastern Region | Aseer | Tabouk | Hail | Northern Borders | Jazan | Najran | Albaha | Aljouf |
|  |  | 91.9 | 88.77 | 92.38 | 91.88 | 94.24 | 90.31 | 93.88 | 90.32 | 76.08 | 89.39 | 97.82 | 94.29 | 88.27 |

Table A3. Correlates of outpatient responsiveness: public healthcare facilities only

|  | immediate attention | dignity | communication | independence | confidentiality | choice | surrounding |
| --- | --- | --- | --- | --- | --- | --- | --- |
|  |  |  |  |  |  |  |  |
| relative to no chronic illness | | | |  |  |  |  |
|  |  |  |  |  |  |  |  |
| 1 chronic illness | 1.088 | 1.360* | 1.153 | 1.023 | 1.041 | 0.772*** | 1.186* |
|  | (0.109) | (0.218) | (0.199) | (0.119) | (0.170) | (0.0707) | (0.105) |
|  |  |  |  |  |  |  |  |
| 2 chronic illnesses | 0.926 | 0.856 | 0.812 | 0.738** | 0.552*** | 0.681*** | 0.965 |
|  | (0.164) | (0.161) | (0.153) | (0.108) | (0.108) | (0.0952) | (0.130) |
|  |  |  |  |  |  |  |  |
| 3 chronic illnesses | 0.802 | 1.126 | 1.037 | 0.623*** | 0.700 | 0.702** | 1.097 |
|  | (0.148) | (0.232) | (0.234) | (0.108) | (0.189) | (0.108) | (0.237) |
|  |  |  |  |  |  |  |  |
| 4 chronic illnesses | 0.740 | 0.901 | 0.699 | 0.638*** | 0.491** | 0.523** | 1.164 |
|  | (0.253) | (0.231) | (0.196) | (0.0946) | (0.158) | (0.133) | (0.428) |
|  |  |  |  |  |  |  |  |
| 5 chronic illnesses | 0.692 | 0.621 | 0.792 | 0.490** | 0.349*** | 0.551** | 0.829 |
|  | (0.158) | (0.207) | (0.235) | (0.137) | (0.111) | (0.130) | (0.307) |
| relative to very bad self rated health | | |  |  |  |  |  |
| bad srh | 2.299** | 2.654** | 2.049 | 1.955 | 2.372 | 1.310 | 2.420* |
|  | (0.909) | (1.303) | (1.035) | (0.891) | (1.488) | (0.593) | (1.123) |
|  |  |  |  |  |  |  |  |
| moderate srh | 2.930*** | 2.335* | 1.698 | 1.438 | 1.509 | 1.648 | 2.362** |
|  | (0.981) | (1.096) | (0.799) | (0.786) | (0.772) | (0.891) | (0.903) |
|  |  |  |  |  |  |  |  |
| good srh | 4.858*** | 4.975*** | 2.593** | 2.410 | 3.188** | 1.708 | 3.800*** |
|  | (1.813) | (2.401) | (1.220) | (1.397) | (1.717) | (0.904) | (1.611) |
|  |  |  |  |  |  |  |  |
| very good srh | 5.941*** | 4.297*** | 3.357** | 2.754 | 2.277 | 2.568* | 3.395*** |
|  | (2.259) | (2.090) | (1.737) | (1.851) | (1.247) | (1.462) | (1.496) |
|  |  |  |  |  |  |  |  |
| female | 1.008 | 1.228 | 0.958 | 1.256** | 1.400* | 1.165 | 0.929 |
|  | (0.0871) | (0.202) | (0.116) | (0.116) | (0.252) | (0.138) | (0.104) |
|  |  |  |  |  |  |  |  |
| relative to less than 20 years old | |  |  |  |  |  |  |
| 21 to 30 | 1.188 | 0.806 | 1.299 | 1.252 | 0.756 | 1.082 | 1.760** |
|  | (0.206) | (0.200) | (0.389) | (0.440) | (0.306) | (0.333) | (0.466) |
|  |  |  |  |  |  |  |  |
| 31 to 40 | 1.160 | 0.836 | 1.248 | 0.933 | 0.809 | 0.845 | 1.459* |
|  | (0.218) | (0.213) | (0.311) | (0.325) | (0.340) | (0.233) | (0.329) |
|  |  |  |  |  |  |  |  |
| 41 to 50 | 1.388 | 1.058 | 1.287 | 1.354 | 0.947 | 1.308 | 1.790** |
|  | (0.285) | (0.256) | (0.329) | (0.484) | (0.428) | (0.411) | (0.421) |
|  |  |  |  |  |  |  |  |
| 51 to 60 | 1.880*** | 1.142 | 1.829** | 1.755 | 1.307 | 1.485 | 1.582* |
|  | (0.441) | (0.354) | (0.477) | (0.680) | (0.554) | (0.461) | (0.377) |
|  |  |  |  |  |  |  |  |
| 61 to 70 | 1.966** | 0.998 | 1.597 | 1.911* | 1.265 | 2.001** | 1.914** |
|  | (0.530) | (0.359) | (0.491) | (0.733) | (0.592) | (0.629) | (0.579) |
|  |  |  |  |  |  |  |  |
| over 71 | 2.765*** | 2.607** | 2.177*** | 2.320** | 1.790 | 2.347** | 2.135** |
|  | (0.668) | (1.273) | (0.614) | (0.859) | (0.783) | (0.844) | (0.793) |
|  |  |  |  |  |  |  |  |
| relative to can't read and write | |  |  |  |  |  |  |
| can read and write | 0.625* | 0.735 | 0.721 | 0.748 | 0.653 | 0.694 | 0.757 |
|  | (0.156) | (0.290) | (0.296) | (0.337) | (0.207) | (0.181) | (0.210) |
|  |  |  |  |  |  |  |  |
| primary completed | 0.739 | 0.870 | 0.803 | 0.791 | 1.229 | 0.812 | 0.550** |
|  | (0.168) | (0.285) | (0.283) | (0.275) | (0.344) | (0.245) | (0.145) |
|  |  |  |  |  |  |  |  |
| middle school completed | 0.733 | 1.051 | 0.615 | 0.856 | 1.157 | 1.047 | 0.560* |
|  | (0.142) | (0.391) | (0.226) | (0.375) | (0.327) | (0.297) | (0.168) |
|  |  |  |  |  |  |  |  |
| high school completed | 0.766 | 0.744 | 0.589 | 0.828 | 0.906 | 0.922 | 0.552** |
|  | (0.185) | (0.263) | (0.221) | (0.353) | (0.198) | (0.261) | (0.154) |
|  |  |  |  |  |  |  |  |
| university | 0.649 | 0.638 | 0.487* | 0.717 | 0.780 | 0.779 | 0.419*** |
|  | (0.173) | (0.259) | (0.205) | (0.327) | (0.217) | (0.247) | (0.134) |
|  |  |  |  |  |  |  |  |
| post-graduate | 0.813 | 3.313 | 0.607 | 1.179 | 2.071 | 1.324 | 0.631 |
|  | (0.290) | (2.504) | (0.291) | (0.529) | (1.197) | (0.519) | (0.352) |
|  |  |  |  |  |  |  |  |
| nationals | 0.513*** | 0.687* | 0.508*** | 0.988 | 0.569* | 1.002 | 0.389*** |
|  | (0.0971) | (0.144) | (0.132) | (0.179) | (0.170) | (0.322) | (0.101) |
|  |  |  |  |  |  |  |  |
| relative to the outpatient centres of the MoH | | |  |  |  |  |  |
| Ministry of interior | 1.765 | 3.267** | 2.317* | 1.926** | 8.102*** | 2.678*** | 5.223*** |
|  | (0.820) | (1.748) | (1.032) | (0.621) | (6.001) | (0.757) | (1.650) |
|  |  |  |  |  |  |  |  |
| Ministry of National Guard | 2.610*** | 2.171 | 2.926** | 1.394 | 1.161 | 1.927*** | 2.094*** |
|  | (0.741) | (1.265) | (1.543) | (0.549) | (0.467) | (0.457) | (0.509) |
|  |  |  |  |  |  |  |  |
| Ministry of Defense | 1.338 | 1.430 | 1.466 | 1.447 | 1.095 | 1.606** | 1.371 |
|  | (0.335) | (0.387) | (0.402) | (0.357) | (0.314) | (0.376) | (0.318) |
|  |  |  |  |  |  |  |  |
| Ministry of Education | 1.635 | 0.537* | 1.873 | 1.237 | 1.728 | 1.108 | 1.864 |
|  | (0.561) | (0.198) | (0.981) | (0.332) | (1.259) | (0.389) | (0.763) |
|  |  |  |  |  |  |  |  |
| King Faisal Hospital | 1.056 | 1.485 | 3.363* | 2.592* | 15.82*** | 1.644 | 3.301** |
|  | (0.290) | (0.990) | (2.196) | (1.261) | (13.15) | (0.595) | (1.616) |
|  |  |  |  |  |  |  |  |
| N | 4217 | 4216 | 4216 | 4215 | 4213 | 4213 | 4213 |
| pseudo R-sq | 0.070 | 0.074 | 0.070 | 0.081 | 0.083 | 0.070 | 0.075 |

Exponentiated coefficients; Standard errors in parentheses

="* p<0.1 ** p<0.05 *** p<0.01"

the models also control for regional dummies (not shown here)

Table A4. Correlates of inpatient responsiveness, objective, public sector only

|  | immediate attention | dignity | communication | independence | confidentiality | choice | surrounding |
| --- | --- | --- | --- | --- | --- | --- | --- |
|  |  |  |  |  |  |  |  |
| relative to no chronic illness | |  |  |  |  |  |  |
| 1 chronic illness | 1.121 | 1.112 | 1.164 | 1.244 | 1.206 | 0.850 | 1.414* |
|  | (0.256) | (0.255) | (0.228) | (0.260) | (0.322) | (0.146) | (0.254) |
|  |  |  |  |  |  |  |  |
| 2 chronic illnesses | 1.147 | 0.901 | 0.895 | 0.795 | 0.966 | 0.652** | 0.946 |
|  | (0.290) | (0.197) | (0.220) | (0.149) | (0.317) | (0.130) | (0.172) |
|  |  |  |  |  |  |  |  |
| 3 chronic illnesses | 0.860 | 0.786 | 1.065 | 0.589* | 0.627 | 0.621*** | 1.016 |
|  | (0.243) | (0.245) | (0.322) | (0.176) | (0.232) | (0.0903) | (0.234) |
|  |  |  |  |  |  |  |  |
| 4 chronic illnesses | 1.138 | 1.039 | 1.380 | 0.521** | 0.826 | 0.591* | 1.047 |
|  | (0.450) | (0.358) | (0.457) | (0.164) | (0.449) | (0.168) | (0.242) |
|  |  |  |  |  |  |  |  |
| 5 chronic illnesses | 1.222 | 0.709 | 0.950 | 0.350*** | 0.574 | 0.713 | 1.526 |
|  | (0.465) | (0.257) | (0.301) | (0.129) | (0.277) | (0.258) | (0.505) |
|  |  |  |  |  |  |  |  |
| relative to very bad self rated health | | |  |  |  |  |  |
| bad srh | 2.833** | 1.601 | 1.884 | 1.236 | 1.843 | 2.264* | 3.254* |
|  | (1.287) | (0.688) | (0.922) | (0.491) | (1.027) | (1.111) | (2.015) |
|  |  |  |  |  |  |  |  |
| moderate srh | 2.390** | 2.419* | 2.097 | 1.420 | 2.107 | 2.341 | 3.304* |
|  | (1.020) | (1.152) | (1.070) | (0.605) | (1.012) | (1.385) | (2.020) |
|  |  |  |  |  |  |  |  |
| good srh | 3.469*** | 3.596*** | 3.546** | 2.351** | 4.989*** | 3.042* | 4.646*** |
|  | (1.268) | (1.694) | (1.852) | (0.941) | (2.457) | (1.737) | (2.707) |
|  |  |  |  |  |  |  |  |
| very good srh | 3.727*** | 2.523** | 3.574*** | 2.670** | 5.089*** | 3.503** | 6.211*** |
|  | (1.571) | (1.128) | (1.649) | (1.259) | (2.708) | (2.189) | (3.841) |
|  |  |  |  |  |  |  |  |
| female | 1.182 | 0.980 | 1.112 | 0.985 | 1.219 | 1.199 | 0.877 |
|  | (0.136) | (0.176) | (0.208) | (0.159) | (0.288) | (0.185) | (0.173) |
|  |  |  |  |  |  |  |  |
| relative to less than 20 years old | |  |  |  |  |  |  |
| 21 to 30 | 1.121 | 1.817 | 1.163 | 0.942 | 2.114 | 1.110 | 1.061 |
|  | (0.393) | (0.832) | (0.506) | (0.459) | (1.041) | (0.424) | (0.456) |
|  |  |  |  |  |  |  |  |
| 31 to 40 | 1.514 | 1.340 | 1.399 | 1.038 | 1.634 | 0.899 | 0.961 |
|  | (0.470) | (0.579) | (0.491) | (0.418) | (0.728) | (0.278) | (0.371) |
|  |  |  |  |  |  |  |  |
| 41 to 50 | 1.668* | 2.267** | 1.676 | 1.649 | 2.758** | 1.211 | 1.059 |
|  | (0.509) | (0.857) | (0.556) | (0.588) | (1.248) | (0.359) | (0.398) |
|  |  |  |  |  |  |  |  |
| 51 to 60 | 1.506 | 1.948* | 1.405 | 1.656 | 2.427* | 1.337 | 1.094 |
|  | (0.513) | (0.774) | (0.490) | (0.736) | (1.113) | (0.488) | (0.379) |
|  |  |  |  |  |  |  |  |
| 61 to 70 | 2.215** | 1.781 | 1.267 | 1.537 | 2.559* | 1.968* | 0.994 |
|  | (0.770) | (0.762) | (0.413) | (0.650) | (1.374) | (0.719) | (0.394) |
|  |  |  |  |  |  |  |  |
| over 71 | 2.356*** | 3.001** | 1.649 | 3.002** | 3.682*** | 1.729 | 1.262 |
|  | (0.782) | (1.455) | (0.679) | (1.330) | (1.849) | (0.784) | (0.621) |
|  |  |  |  |  |  |  |  |
| relative to can't read and write | |  |  |  |  |  |  |
| can read and write | 0.885 | 0.748 | 0.793 | 0.567 | 1.165 | 0.635 | 1.124 |
|  | (0.246) | (0.284) | (0.284) | (0.230) | (0.428) | (0.185) | (0.343) |
|  |  |  |  |  |  |  |  |
| primary completed | 0.860 | 0.590 | 0.797 | 0.574 | 1.395 | 0.690 | 0.713 |
|  | (0.158) | (0.197) | (0.245) | (0.220) | (0.677) | (0.186) | (0.201) |
|  |  |  |  |  |  |  |  |
| middle school completed | 1.116 | 0.743 | 0.821 | 0.528* | 1.152 | 0.915 | 0.670 |
|  | (0.356) | (0.277) | (0.299) | (0.201) | (0.476) | (0.286) | (0.253) |
|  |  |  |  |  |  |  |  |
| high school completed | 0.965 | 0.819 | 0.742 | 0.653 | 1.190 | 0.864 | 0.638 |
|  | (0.209) | (0.279) | (0.273) | (0.241) | (0.487) | (0.254) | (0.198) |
|  |  |  |  |  |  |  |  |
| university | 0.747 | 0.598 | 0.506* | 0.394** | 0.633 | 0.577* | 0.390** |
|  | (0.210) | (0.243) | (0.207) | (0.175) | (0.305) | (0.188) | (0.143) |
|  |  |  |  |  |  |  |  |
| post-graduate | 1.192 | 2.594 | 1.331 | 1.213 | 1 | 1.271 | 0.589 |
|  | (0.596) | (2.285) | (1.131) | (1.220) | (.) | (0.774) | (0.488) |
|  |  |  |  |  |  |  |  |
| nationals | 0.483*** | 0.503* | 0.577 | 1.199 | 0.560 | 0.866 | 0.525* |
|  | (0.108) | (0.196) | (0.196) | (0.310) | (0.231) | (0.239) | (0.176) |
|  |  |  |  |  |  |  |  |
| relative to hospitals belonging to MoH | | |  |  |  |  |  |
| Ministry of Interior | 4.361* | 4.954 | 1 | 2.854 | 2.671 | 1.063 | 2.801* |
|  | (3.327) | (5.505) | (.) | (2.325) | (2.656) | (0.590) | (1.649) |
|  |  |  |  |  |  |  |  |
| Ministry of National Guard | 1.956 | 2.759 | 4.643*** | 3.350* | 2.114 | 1.769* | 5.289** |
|  | (1.120) | (2.167) | (2.676) | (2.094) | (1.858) | (0.557) | (3.952) |
|  |  |  |  |  |  |  |  |
| Ministry of Defense | 2.294** | 1.778 | 3.227** | 2.330** | 2.740* | 1.628* | 2.614** |
|  | (0.970) | (0.695) | (1.821) | (0.794) | (1.444) | (0.441) | (0.988) |
|  |  |  |  |  |  |  |  |
| Ministry of Education | 1.255 | 1.208 | 2.314 | 2.619 | 3.165 | 1.502 | 1.236 |
|  | (0.422) | (0.551) | (1.863) | (2.112) | (2.658) | (0.910) | (0.892) |
|  |  |  |  |  |  |  |  |
| King Faisal Hospital | 4.087** | 5.772** | 7.927* | 5.087** | 5.952** | 2.290* | 8.821*** |
|  | (2.514) | (4.931) | (8.707) | (3.897) | (5.039) | (0.974) | (6.648) |
|  |  |  |  |  |  |  |  |
| N | 1811 | 1810 | 1782 | 1810 | 1606 | 1809 | 1809 |
| pseudo R-sq | 0.071 | 0.081 | 0.070 | 0.101 | 0.097 | 0.068 | 0.086 |

Exponentiated coefficients; Standard errors in parentheses

="* p<0.1 ** p<0.05 *** p<0.01"

the models also control for regional dummies (not reported here)

Table A5. Index of outpatient and inpatient responsiveness, objective

|  | outpatient responsiveness index | inpatient responsiveness index |  |
| --- | --- | --- | --- |
|  |  |  |  |
| relative to no chronic illnesses | |  |  |
| 1 chronic illness | 1.284** | 0.804 |  |
|  | (0.153) | (0.161) |  |
|  |  |  |  |
| 2 chronic illnesses | 0.785 | 0.656* |  |
|  | (0.119) | (0.148) |  |
|  |  |  |  |
| 3 chronic illnesses | 0.911 | 0.467** |  |
|  | (0.202) | (0.139) |  |
|  |  |  |  |
| 4 chronic illnesses | 0.807 | 0.516** |  |
|  | (0.282) | (0.174) |  |
|  |  |  |  |
| 5 chronic illnesses | 0.542** | 0.327*** |  |
|  | (0.163) | (0.108) |  |
|  |  |  |  |
| relative to very bad self rated health | |  |  |
| bad srh | 2.148 | 1.358 |  |
|  | (1.160) | (0.637) |  |
|  |  |  |  |
| moderate srh | 2.425* | 1.684 |  |
|  | (1.287) | (0.912) |  |
|  |  |  |  |
| good srh | 4.322*** | 3.748*** |  |
|  | (1.938) | (1.881) |  |
|  |  |  |  |
| very good srh | 5.013*** | 3.105** |  |
|  | (2.544) | (1.459) |  |
|  |  |  |  |
| female | 1.194 | 1.416** |  |
|  | (0.134) | (0.250) |  |
|  |  |  |  |
| relative to less than 20 years old | |  |  |
| 21 to 30 | 1.032 | 0.947 |  |
|  | (0.292) | (0.494) |  |
|  |  |  |  |
| 31 to 40 | 1.123 | 1.246 |  |
|  | (0.293) | (0.502) |  |
|  |  |  |  |
| 41 to 50 | 1.592 | 1.802* |  |
|  | (0.525) | (0.623) |  |
|  |  |  |  |
| 51 to 60 | 2.162** | 1.868* |  |
|  | (0.819) | (0.693) |  |
|  |  |  |  |
| 61 to 70 | 2.137* | 2.700** |  |
|  | (0.934) | (1.246) |  |
|  |  |  |  |
| over 71 | 3.195*** | 4.606*** |  |
|  | (1.381) | (1.963) |  |
|  |  |  |  |
| relative to can't read and write | |  |  |
| can read and write | 0.627 | 0.886 |  |
|  | (0.251) | (0.381) |  |
|  |  |  |  |
| primary completed | 0.753 | 0.972 |  |
|  | (0.215) | (0.385) |  |
|  |  |  |  |
| middle school completed | 0.636 | 0.863 |  |
|  | (0.219) | (0.425) |  |
|  |  |  |  |
| high school completed | 0.682 | 1.065 |  |
|  | (0.204) | (0.495) |  |
|  |  |  |  |
| university | 0.712 | 0.619 |  |
|  | (0.283) | (0.308) |  |
|  |  |  |  |
| post-graduate | 1.622 | 0.705 |  |
|  | (1.077) | (0.534) |  |
|  |  |  |  |
| nationals | 0.562** | 0.710* |  |
|  | (0.127) | (0.124) |  |
|  |  |  |  |
| public outpatient centre | 0.480*** |  |  |
|  | (0.0731) |  |  |
|  |  |  |  |
| public hospital |  | 0.246*** |  |
|  |  | (0.0585) |  |
|  |  |  |  |
| N | 6840 | 2031 |  |
| pseudo R-sq | 0.146 | 0.144 |  |

Exponentiated coefficients; Standard errors in parentheses

="* p<0.1 ** p<0.05 *** p<0.01"

the models also control for regional dummies, not reportedhere

Table A6. Index of inpatient responsiveness, subjective

|  | index of outpatient responsiveness | index of inpatient responsiveness |
| --- | --- | --- |
|  |  |  |
| Have you been treated badly because of |  |  |
| nationality | 1.351 | 0.531 |
|  | (0.497) | (0.240) |
|  |  |  |
| social background | 0.336*** | 0.373** |
|  | (0.116) | (0.179) |
|  |  |  |
| lack of insurance | 0.773 | 0.300*** |
|  | (0.198) | (0.0770) |
|  |  |  |
| skin colour | 0.324*** | 0.571 |
|  | (0.106) | (0.391) |
|  |  |  |
| gender | 0.987 | 1.420 |
|  | (0.341) | (0.478) |
|  |  |  |
| language | 0.756 | 0.781 |
|  | (0.237) | (0.369) |
|  |  |  |
| religion | 2.561** | 0.952 |
|  | (1.184) | (0.802) |
|  |  |  |
| health status | 0.504** | 1.349 |
|  | (0.170) | (0.589) |
|  |  |  |
| weak physical conditions | 0.619*** | 0.892 |
|  | (0.114) | (0.380) |
| N | 6908 | 2054 |
| pseudo R-sq | 0.142 | 0.137 |

Exponentiated coefficients; Standard errors in parentheses

="* p<0.1 ** p<0.05 *** p<0.01"

the models also control for nationality, regional dummies and place of seeking care (not shown here)

**The RECORD statement – checklist of items, extended from the STROBE statement, that should be reported in observational studies using routinely collected health data.**

|  | **Item No.** | **STROBE items** | **Location in manuscript where items are reported** | **RECORD items** | **Location in manuscript where items are reported** |
| --- | --- | --- | --- | --- | --- |
| **Title and abstract** | | | | | |
|  | 1 | (a) Indicate the study’s design with a commonly used term in the title or the abstract (b) Provide in the abstract an informative and balanced summary of what was done and what was found | (a) Title of the manuscript  (b) sections: background, methods, results and conclusion in the abstract of the manuscript. | RECORD 1.1: The type of data used should be specified in the title or abstract. When possible, the name of the databases used should be included.  RECORD 1.2: If applicable, the geographic region and timeframe within which the study took place should be reported in the title or abstract.  RECORD 1.3: If linkage between databases was conducted for the study, this should be clearly stated in the title or abstract. |  |
| **Introduction** | | | | | |
| Background rationale | 2 | Explain the scientific background and rationale for the investigation being reported | Introduction, paragraph 1, 2, 3, 4 and 5 |  |  |
| Objectives | 3 | State specific objectives, including any prespecified hypotheses | Introduction, paragraph 7 and 8 |  |  |
| **Methods** | | | | | |
| Study Design | 4 | Present key elements of study design early in the paper | Para 1 and 2 in the methods |  |  |
| Setting | 5 | Describe the setting, locations, and relevant dates, including periods of recruitment, exposure, follow-up, and data collection | Para 1 in the methods (section data) |  |  |
| Participants | 6 | *(a) Cohort study* - Give the eligibility criteria, and the sources and methods of selection of participants. Describe methods of follow-up  *Case-control study* - Give the eligibility criteria, and the sources and methods of case ascertainment and control selection. Give the rationale for the choice of cases and controls  *Cross-sectional study* - Give the eligibility criteria, and the sources and methods of selection of participants  *(b) Cohort study* - For matched studies, give matching criteria and number of exposed and unexposed  *Case-control study* - For matched studies, give matching criteria and the number of controls per case | Para 1 (section data) | RECORD 6.1: The methods of study population selection (such as codes or algorithms used to identify subjects) should be listed in detail. If this is not possible, an explanation should be provided.  RECORD 6.2: Any validation studies of the codes or algorithms used to select the population should be referenced. If validation was conducted for this study and not published elsewhere, detailed methods and results should be provided.  RECORD 6.3: If the study involved linkage of databases, consider use of a flow diagram or other graphical display to demonstrate the data linkage process, including the number of individuals with linked data at each stage. |  |
| Variables | 7 | Clearly define all outcomes, exposures, predictors, potential confounders, and effect modifiers. Give diagnostic criteria, if applicable. | Para 2, 3, 4, 5 and 6 (section dependent and independent variables) | RECORD 7.1: A complete list of codes and algorithms used to classify exposures, outcomes, confounders, and effect modifiers should be provided. If these cannot be reported, an explanation should be provided. |  |
| Data sources/ measurement | 8 | For each variable of interest, give sources of data and details of methods of assessment (measurement).  Describe comparability of assessment methods if there is more than one group | Para 2, 3, 4, 5 and 6 (section dependent and independent variables) |  |  |
| Bias | 9 | Describe any efforts to address potential sources of bias | Para 8 in the methods section |  |  |
| Study size | 10 | Explain how the study size was arrived at | Para 1 (section data) |  |  |
| Quantitative variables | 11 | Explain how quantitative variables were handled in the analyses. If applicable, describe which groupings were chosen, and why | Para 2, 3, 4, 5 and 6 (section dependent and independent variables) |  |  |
| Statistical methods | 12 | (a) Describe all statistical methods, including those used to control for confounding  (b) Describe any methods used to examine subgroups and interactions  (c) Explain how missing data were addressed  (d) *Cohort study* - If applicable, explain how loss to follow-up was addressed  *Case-control study* - If applicable, explain how matching of cases and controls was addressed  *Cross-sectional study* - If applicable, describe analytical methods taking account of sampling strategy  (e) Describe any sensitivity analyses | Para 2, 3, 4, 5, 6, 7 and 8 (section dependent and independent variables) |  |  |
| Data access and cleaning methods |  | .. |  | RECORD 12.1: Authors should describe the extent to which the investigators had access to the database population used to create the study population.  RECORD 12.2: Authors should provide information on the data cleaning methods used in the study. |  |
| Linkage |  | .. |  | RECORD 12.3: State whether the study included person-level, institutional-level, or other data linkage across two or more databases. The methods of linkage and methods of linkage quality evaluation should be provided. |  |
| **Results** | | | | | |
| Participants | 13 | (a) Report the numbers of individuals at each stage of the study (*e.g.*, numbers potentially eligible, examined for eligibility, confirmed eligible, included in the study, completing follow-up, and analysed)  (b) Give reasons for non-participation at each stage.  (c) Consider use of a flow diagram | Para 1 and 2 in the results section | RECORD 13.1: Describe in detail the selection of the persons included in the study (*i.e.,* study population selection) including filtering based on data quality, data availability and linkage. The selection of included persons can be described in the text and/or by means of the study flow diagram. |  |
| Descriptive data | 14 | (a) Give characteristics of study participants (*e.g.*, demographic, clinical, social) and information on exposures and potential confounders  (b) Indicate the number of participants with missing data for each variable of interest  (c) *Cohort study* - summarise follow-up time (*e.g.*, average and total amount) | Para 1 and 2 in the results section |  |  |
| Outcome data | 15 | *Cohort study* - Report numbers of outcome events or summary measures over time  *Case-control study* - Report numbers in each exposure category, or summary measures of exposure  *Cross-sectional study* - Report numbers of outcome events or summary measures | Para 1 and 2 in the results section |  |  |
| Main results | 16 | (a) Give unadjusted estimates and, if applicable, confounder-adjusted estimates and their precision (e.g., 95% confidence interval). Make clear which confounders were adjusted for and why they were included  (b) Report category boundaries when continuous variables were categorized  (c) If relevant, consider translating estimates of relative risk into absolute risk for a meaningful time period | Para 3, 4, 5, 6 and 7 in the results section |  |  |
| Other analyses | 17 | Report other analyses done—e.g., analyses of subgroups and interactions, and sensitivity analyses | Para 8, 9, 10 and 11 in the results section |  |  |
| **Discussion** | | | | | |
| Key results | 18 | Summarise key results with reference to study objectives | Para 1 in the discussion section |  |  |
| Limitations | 19 | Discuss limitations of the study, taking into account sources of potential bias or imprecision. Discuss both direction and magnitude of any potential bias | Para 5 in the discussion section | RECORD 19.1: Discuss the implications of using data that were not created or collected to answer the specific research question(s). Include discussion of misclassification bias, unmeasured confounding, missing data, and changing eligibility over time, as they pertain to the study being reported. |  |
| Interpretation | 20 | Give a cautious overall interpretation of results considering objectives, limitations, multiplicity of analyses, results from similar studies, and other relevant evidence | Para 2, 3 and 4 in the discussion section |  |  |
| Generalisability | 21 | Discuss the generalisability (external validity) of the study results | Para 5 in the discussion section |  |  |
| **Other Information** | | | | | |
| Funding | 22 | Give the source of funding and the role of the funders for the present study and, if applicable, for the original study on which the present article is based | Info provided at both, abstract and end of manuscript |  |  |
| Accessibility of protocol, raw data, and programming code |  | .. |  | RECORD 22.1: Authors should provide information on how to access any supplemental information such as the study protocol, raw data, or programming code. |  |

*Reference: Benchimol EI, Smeeth L, Guttmann A, Harron K, Moher D, Petersen I, Sørensen HT, von Elm E, Langan SM, the RECORD Working Committee. The REporting of studies Conducted using Observational Routinely-collected health Data (RECORD) Statement. *PLoS Medicine* 2015; in press.

*Checklist is protected under Creative Commons Attribution ([CC BY](http://creativecommons.org/licenses/by/4.0/)) license.
